# A Data-Driven Algorithm to Recommend Initial Clinical Workup for Outpatient Specialty Referral: Algorithm Development and Validation Using Electronic Health Record Data and Expert Surveys

**DOI:** 10.2196/30104

**Published:** 2022-03-03

**Authors:** Wui Ip, Priya Prahalad, Jonathan Palma, Jonathan H Chen

**Affiliations:** 1 Department of Pediatrics Stanford University School of Medicine Palo Alto, CA United States; 2 Neonatology & Perinatal Medicine Orlando Health Winnie Palmer Hospital for Women & Babies Orlando, FL United States; 3 Department of Medicine Stanford University School of Medicine Palo Alto, CA United States; 4 Stanford Center for Biomedical Informatics Research Stanford, CA United States

**Keywords:** recommender system, electronic health records, clinical decision support, specialty consultation, machine learning, EHR, algorithm, algorithm development, algorithm validation, automation, prediction, patient needs

## Abstract

**Background:**

Millions of people have limited access to specialty care. The problem is exacerbated by ineffective specialty visits due to incomplete prereferral workup, leading to delays in diagnosis and treatment. Existing processes to guide prereferral diagnostic workup are labor-intensive (ie, building a consensus guideline between primary care doctors and specialists) and require the availability of the specialists (ie, electronic consultation).

**Objective:**

Using pediatric endocrinology as an example, we develop a recommender algorithm to anticipate patients’ initial workup needs at the time of specialty referral and compare it to a reference benchmark using the most common workup orders. We also evaluate the clinical appropriateness of the algorithm recommendations.

**Methods:**

Electronic health record data were extracted from 3424 pediatric patients with new outpatient endocrinology referrals at an academic institution from 2015 to 2020. Using item co-occurrence statistics, we predicted the initial workup orders that would be entered by specialists and assessed the recommender’s performance in a holdout data set based on what the specialists actually ordered. We surveyed endocrinologists to assess the clinical appropriateness of the predicted orders and to understand the initial workup process.

**Results:**

Specialists (n=12) indicated that <50% of new patient referrals arrive with complete initial workup for common referral reasons. The algorithm achieved an area under the receiver operating characteristic curve of 0.95 (95% CI 0.95-0.96). Compared to a reference benchmark using the most common orders, precision and recall improved from 37% to 48% (*P*<.001) and from 27% to 39% (*P*<.001) for the top 4 recommendations, respectively. The top 4 recommendations generated for common referral conditions (abnormal thyroid studies, obesity, amenorrhea) were considered clinically appropriate the majority of the time by specialists surveyed and practice guidelines reviewed.

**Conclusions:**

An item association–based recommender algorithm can predict appropriate specialists’ workup orders with high discriminatory accuracy. This could support future clinical decision support tools to increase effectiveness and access to specialty referrals. Our study demonstrates important first steps toward a data-driven paradigm for outpatient specialty consultation with a tier of automated recommendations that proactively enable initial workup that would otherwise be delayed by awaiting an in-person visit.

## Introduction

### Background

There is a fundamental and growing gap between the supply and demand of medical expertise, as reflected in the projected shortage of 100,000 physicians by 2030 [1]. The problem is particularly acute for specialty care [2-6], for which over 25 million people in the United States have deficient access [7]. Wait times for in-person specialty visits commonly extend weeks to months after referrals are made [5]. Adding to this problem, essential initial workup is often not completed [8,9], resulting in ineffective visits when the specialists do not have sufficient information to make a definitive diagnosis and treatment recommendations by the time of their first in-person visit. Such inefficiency could lead to care delay, missed opportunity to provide access to more patients, and dissatisfaction of patients and families.

Ideally, referring providers could directly communicate with specialists for their preconsultation advice on an initial recommended clinical workup. However, data show that primary care providers are only able to communicate with specialists half of the time when referring patients [10]. Alternatively, primary care providers and specialists can collaboratively develop guidelines for initial workup [11], but this requires substantial manual effort to produce and maintain up-to-date content as new evidence arises and practice changes over time. Asynchronous electronic consults or synchronous telemedicine consults are emerging approaches for referring providers to solicit specialists’ opinions on the need of referral and initial workup [12-16], with potential advantages of streamlining the referral process and empowering primary care providers. However, such consults remain limited in availability, as they still require a human consultant to review and respond to each request [17,18].

A more data-driven approach could boost the capacity of the health system by making initial specialty clinic visits more effective and by sparing the time required by specialists to communicate initial workup needs. Prior studies have shown the efficacy of statistical approaches, including association rules, Bayesian networks, logistic regression, and deep neural networks, for generating clinical order recommendations. The focus of these studies, however, has been primarily in the acute care settings such as inpatient hospitalization and emergency room visits [19-26].

Our aim is to develop a data-driven paradigm for outpatient specialty consultation with a tier of automated recommendations that proactively enable initial workup that would otherwise be delayed by awaiting an in-person visit. Taking advantage of electronic health records that contain thousands of specialist referral visits, we propose a data-driven algorithm inspired by Amazon’s “customers who bought A also bought B” [27] to anticipate initial specialty evaluations at the time of referral based on how specialists cared for similar patients in the past. In this study, we chose pediatric endocrinology as a use case because laboratory evaluation is often required to inform specialist treatment recommendations [28-30].

### Objective

Using specialty referrals to pediatric endocrinology as an example, we developed a recommender algorithm to anticipate initial workup needs for a variety of endocrine conditions. We compared the performance of the algorithm to a reference benchmark based upon the most common workup orders. We evaluated the need to complete initial workup and the clinical appropriateness of the algorithm recommendations by surveying specialists.

## Methods

### Recommender Algorithm Development

Deidentified structured electronic health record data from outpatient clinic visits at Stanford Children’s Health were extracted from the Stanford Medicine Research Data Repository using the Observational Medical Outcomes Partnership (OMOP) common data model [31]. We include patients younger than 18 years with a pediatric endocrine referral order from any Stanford-affiliated clinic and a subsequent pediatric endocrine visit within 6 months. Between 2015 and 2020, 3424 patients met criteria, whose data yielded >1,150,000 instances of 8263 distinct clinical items.

We used OMOP common data model concepts to define distinct clinical items, including 2966 conditions, 2423 measurements (eg, lab results), 1187 procedures (eg, diagnostic imaging), and 1687 medications. Numeric laboratory results were categorized as “normal,” “high,” or “low” based upon reference ranges. We excluded clinical items that occurred in fewer than 10 patients.

**Figure 1:**
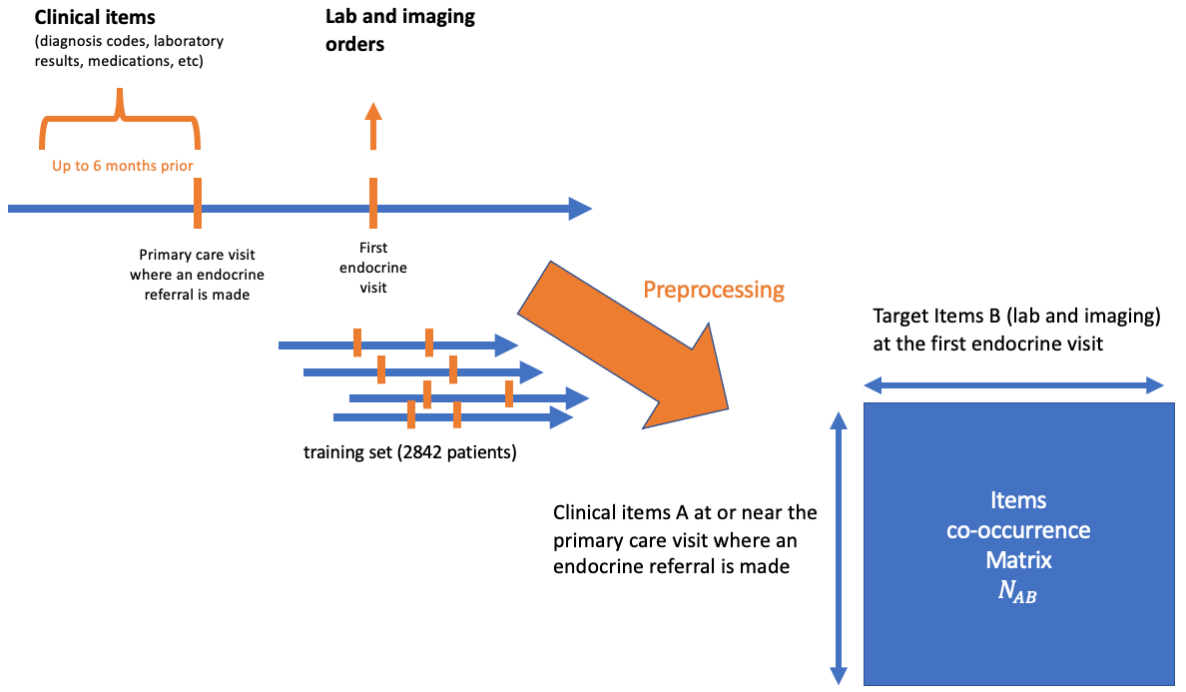
Algorithm training and construction of the item co-occurrence matrix.

Based on the timing of pediatric endocrinology referral, we split the patient cohort into a training set (referrals from 2015 to 2019: n=2842 patients) and a test set (referral in 2020: n=582 patients). In the training set, we calculated the co-occurrence statistics of pairs of clinical items to build an item association matrix (Figure 1). We counted duplicate items only once per patient to allow natural interpretation of patient prevalence and diagnostic measures.

The recommender algorithm is queried with a patient’s clinical items (diagnosis, labs, medications, etc) associated with the primary care encounter when the endocrine referral was placed. In addition, we included clinical items associated with the patient in the 6 months prior to the primary care encounter.

Using these clinical items (A_1_,..., A_q_), the recommender algorithm retrieves scores that resemble posttest probability from the co-occurrence association matrix for all possible target items at the subsequent endocrine visits. We limited the target items to laboratory and imaging orders to focus on diagnostic workup recommendations. For each query item (A), target items (B) are ranked by estimated posttest probability P(B|A), or positive predictive value (PPV), defined as the number of patients who have query item A followed by target item B (*N*_AB_) divided by the number of patients with query item A (*N*_A_).




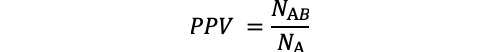




If a patient has *q* query items, *q* separate ranked lists are generated. To aggregate these results, we estimate total pseudo-counts using the following equation that sums across every *i*-th query item:




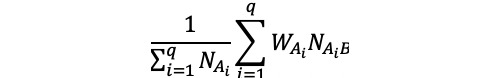




W_A_ is a weighting factor for the query item. There are several ways one can model W_A._ For instance, one can penalize common query items by the following expression:




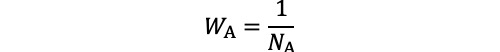




Another method, inspired by a weighting strategy using item clustering based on genres [32], is to weigh a query item based on its relevance to the endocrine referral cohort by using a relative risk term:

*W*_A_=*RR*_A_

Where:




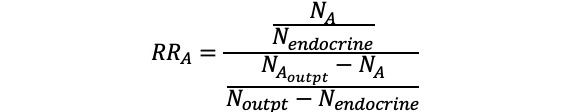




The numerator is the prevalence of item A in our endocrine cohort (*N_endocrine_* is the total number of patients in our endocrine referral cohort, of which *N_A_* patients have clinical item A). The denominator is the prevalence of item A outside of the endocrine cohort in all outpatient clinics (*N_outpt_* is the total number of pediatric patients in all outpatient clinics, of which 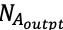 patients have item A).

Using 10-fold cross-validation in our training set, we evaluated these two strategies to model *W_A_* individually and in combination (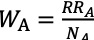). We selected the *W_A_* that gave the best prediction performance in the training data and subsequently used it in the test set.

The code can be accessed via GitHub [33].

### Evaluation Using Electronic Health Record Test Set Data

**Figure 2:**
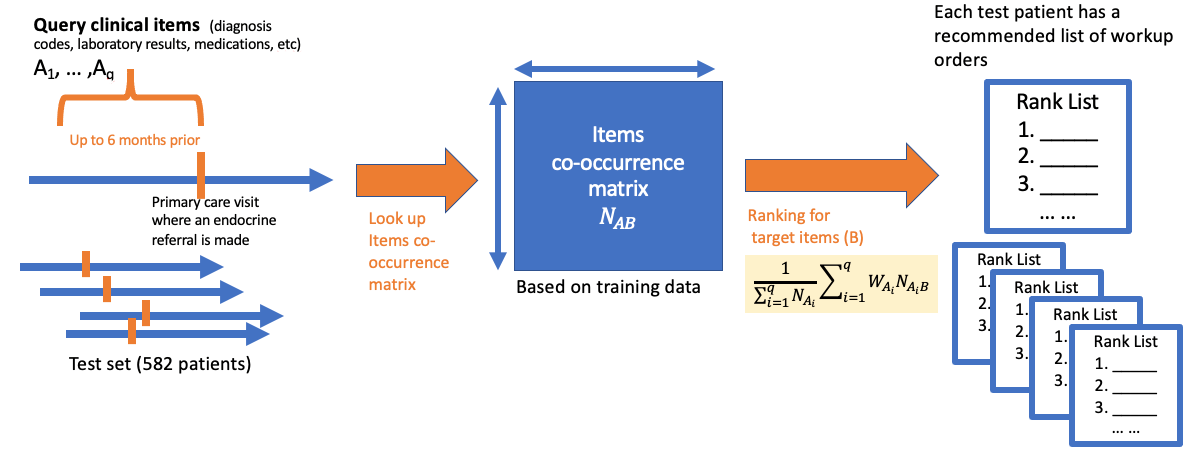
Algorithm evaluation using the test set.

To evaluate the performance of the recommender algorithm in the test set (Figure 2), we compared the recommended list of orders with the actual workup orders patients received at their first endocrine visit. We calculated the precision (PPV) and recall (sensitivity) for the top 4 recommendations, and performed the receiver operating characteristics analysis. We chose the top 4 recommendations because 4 is the mean number of workup orders at the first endocrine visit. We calculated 95% CIs using 1000 bootstrap resamples [34].

### Evaluation Using Expert Surveys

**Figure 3:**
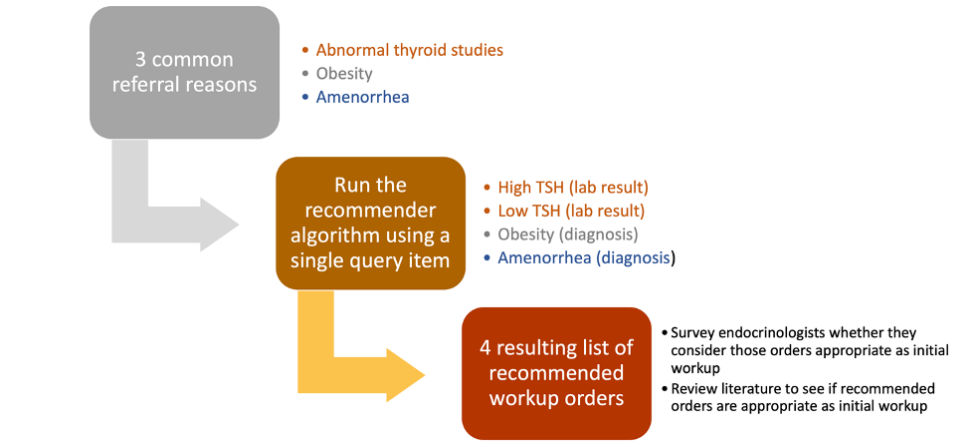
Evaluation of algorithm output by practicing endocrinologists. TSH: thyroid stimulating hormone.

To further understand whether the recommendations would be as clinically appropriate as the initial workup ordered by referring providers, we conducted a survey of all pediatric endocrinologists at Stanford Children’s Health on three common referral reasons (abnormal thyroid studies, obesity, and amenorrhea). The survey was approved by the Institutional Review Board at Stanford University. Survey invitations were sent via emails in July 2020, and survey questions and informed consent were included in the supplemental material. For abnormal thyroid studies, we generated two lists of the top recommended workup orders—one queried with high thyroid stimulating hormone (TSH; an abnormal lab result suggesting hypothyroidism) and the other queried with low TSH (an abnormal lab result suggesting hyperthyroidism). For obesity and amenorrhea, we generated a list of the top recommended orders using the diagnosis as a single query item (Figure 3). Subsequently, we asked the endocrinologists to select the orders from the recommended lists that they considered clinically appropriate as initial workup for the corresponding condition. Other than the referral reasons, the endocrinologists received no other information related to the patients. We instructed them to define appropriate workup as workup that gives sufficient information for the endocrinologists to make concrete recommendations at the clinic visits. In addition, for each of the three conditions, we asked them how often initial workup is completed in their practice and how helpful it is if initial workup is completed prior to the first specialty visit. Lastly, we reviewed published literature and consensus guidelines [28,30,35-37] as external validation to assess whether the recommended orders represent a reasonable workup.

## Results

### Evaluation Using Electronic Health Record Test Set Data

Table 1 compares the performance of the recommender algorithm with a reference benchmark using the most common orders in our endocrine referral cohort (endocrine prevalence). The recommender algorithm had the best performance with an area under the receiver operating characteristic curve (AUC) of 0.95 (95% CI 0.95-0.96). Comparing with the reference benchmark, precision improved from 37% to 48% (*P*<.001), and recall improved from 27% to 39% (*P*<.001). The recommender algorithm is based on a weighting factor, 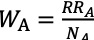, that resulted in the best cross-validation performance in our training data (Multimedia Appendix 1, Table S1).

**Table 1:** Recommender algorithm performance in the test set. Precision and recall were calculated at k=4, given that 4 is the average number of workup orders.

	Recommender^a^	Endocrine prevalence^b^	Outpatient prevalence^c^	Random^d^
Precision^e^ (%; 95% CI)	48 (45-52)	37 (34-40)	10 (8-12)	2 (2-3)
Recall^f^ (%; 95% CI)	39 (36-42)	27 (24-29)	5 (4-6)	2 (1-3)
AUC^g^ (95% CI)	0.95 (0.95-0.96)	0.88 (0.87-0.89)	0.64 (0.62-0.66)	0.49 (0.47-0.5)

^a^Recommender: ranking workup orders using the recommender algorithm.

^b^Endocrine prevalence: ranking workup orders using the percentage of patients who had the orders in the endocrine referral cohort (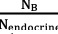) training set.

^c^Outpatient prevalence: ranking workup orders using the percentage of patients who had the orders among all outpatients (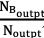).

^d^Random: random ranking of workup orders.

^e^Precision: positive predictive value (proportion of predictions that were correct).

^f^Recall: sensitivity (proportion of correct items that were predicted).

^g^AUC: area under the receiver operating characteristic curve.

### Evaluation Using Expert Surveys

Of 14 pediatric endocrinologists at Stanford Children’s Health, 12 (86%) responded to our survey on three common referral reasons (abnormal thyroid studies, obesity, and amenorrhea). Table 2 shows less than half of the patients coming to the specialty clinics with appropriate initial workup as estimated by the pediatric endocrinologists for each of the three referral reasons (each endocrinologist provided a value between 0% and 100%). The endocrinologists considered it as moderately to very helpful to have the initial workup completed prior to specialty visits (Table 2).

Table 3 shows the top recommendations based on the recommender algorithm using a single query item as mentioned in Figure 3. Each recommended workup order has a corresponding survey result showing the percentage of endocrinologists who considered the order clinically appropriate as the initial workup. Overall, the majority of the specialists considered the top four recommendations clinically appropriate in each of the lists.

**Table 2:** Estimated percentages of patients with initial workup completed before specialty visits and mean Likert scale score of how helpful (5: extremely helpful; 1: not helpful at all) it is to have initial workup completed before specialty visits.

		Value, mean (SD)
**Estimated percentages of patients with initial workup completed before specialty visits (%)**		
	Abnormal thyroid studies	49 (21)
	Obesity	45 (20)
	Amenorrhea	37 (18)
**Likert scale score of how helpful it is to have initial workup completed before specialty visits**		
	Abnormal thyroid studies	4.2 (0.8)
	Obesity	3.3 (0.9)
	Amenorrhea	4.1 (0.8)

**Table 3:** Top recommendations for patients referred for high thyroid stimulating hormone (TSH; commonly due to hypothyroidism), low TSH (commonly due to hypothyroidism), obesity, and amenorrhea.

Orders		PPV^a^ (%)	Relative ratio^b^	Endocrine prevalence^c^ (%)	Outpatient prevalence^d^ (%)	Percent of endocrinologist considered appropriate
**High TSH^e^**						
	TSH^f^	60.9	1.0	61.7	17.1	92
	Free thyroxine^f^	60.3	1.2	56.8	7.5	100
	Thyroglobulin antibody^f^	41.7	3.7	12.8	0.5	92
	Thyroperoxidase antibody^f^	39.1	3.2	13.7	1.0	92
	Vitamin D level	9.3	0.3	31.4	8.1	0
	Serum cortisol	8.6	0.7	11.3	0.7	0
**Low TSH^g^**						
	TSH^h^	61.5	1.0	61.7	17.1	75
	Free thyroxine^h^	57.7	1.0	56.8	7.5	100
	Thyroglobulin antibody^h^	50.0	4.0	12.8	0.5	67
	Thyroperoxidase antibody^h^	46.2	3.4	13.7	1.0	58
	Total tri-iodothyronine^h^	42.3	16.1	3.0	0.7	92
	Comprehensive metabolic panel	26.9	0.5	53.8	23.1	8
**Obesity^i^**						
	Hemoglobin A_1c_^j^	40.2	1.9	22.5	12.5	100
	TSH	28.0	0.4	61.7	17.1	75
	Free thyroxine	25.6	0.4	56.8	7.5	42
	Comprehensive metabolic panel^j^	25.6	0.5	53.8	23.1	92
	Lipid panel^j^	25.0	1.4	18.3	12.9	100
	Vitamin D level	20.7	0.6	31.4	8.1	42
**Amenorrhea^k^**						
	Prolactin^l^	41.4	4.8	8.9	0.4	92
	Luteinizing hormone^l^	37.9	2.2	17.5	0.9	100
	Follicle stimulating hormone^l^	34.5	2.1	16.5	1.7	100
	Estradiol	27.6	4.7	6.1	0.4	100
	17 Hydroxy-progesterone	24.1	2.5	9.7	0.2	100
	Dehydroepi-androsterone sulfate	17.2	2.2	8.0	0.4	92

^a^PPV: positive predictive value.

^b^Relative ratio: the ratio of the probability of the order given the query item to the probability of the order given the lack of the query item.

^c^Endocrine prevalence: the percentage of patients who had the orders in the endocrine referral cohort (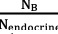).

^d^Outpatient prevalence: the percentage of patients who had the orders among all outpatients (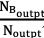).

^e^The top four orders are considered clinically appropriate by almost all of the endocrinologists and are recommended based on published guidelines. The fifth and sixth recommended items have relatively low PPV.

^f^Recommended based on guidelines [36].

^g^Here, the top five orders are considered clinically appropriate by most endocrinologists and published guidelines.

^h^Recommended based on guidelines [37].

^i^Hemoglobin A_1c_, lipid panel, and comprehensive metabolic panel are considered clinically appropriate both by the endocrinologists and published guidelines.

^j^Recommended based on guidelines [35].

^k^The top six orders are considered clinically appropriate by almost all of the endocrinologists. The top three are also recommended based on published literature.

^l^Recommended based on published literature [30].

## Discussion

### Significance

Using pediatric endocrinology as an example, we developed and evaluated a recommender algorithm to anticipate initial workup needs at the time of specialty referral. The algorithm can predict appropriate specialist’s workup orders with high discriminatory accuracy with an AUC>0.9. Our survey shows that, among the three common referral reasons, less than half of the patients typically have appropriate initial workup prior to their initial specialist visit. Most specialists agree that having initial workup completed prior to the first clinic visit is helpful and that our algorithm recommendations for the three referral conditions are clinically appropriate. This supports the potential utility of a data-driven recommender algorithm for referring providers. Although we illustrated 3 common referral conditions in this study, the algorithmic approach is general, and it could be broadly applied to other referral reasons or other specialties, with the benefit of personalization based on individual patient patterns of clinical items, including the combination of multiple conditions.

Although this algorithm is not suitable for full automation given the level of precision and recall, such an algorithm could serve as a clinical decision support tool [38-41] by displaying relevant clinical orders for referring providers to make the referral process more effective. One can imagine coupling this clinical decision support tool with electronic consultation so that specialists can quickly confirm the workup orders in the recommended list, thus augmenting the efficiency of the specialists and increasing their capacity to care for more patients. Advantages of an algorithmic decision support tool compared to building consensus guidelines among specialists [11,42] include scalability to answer unlimited queries on demand, maintainability through automated statistical learning, adaptability to respond to evolving clinical practices [43], and personalizability of individual suggestions with greater accuracy than manually authored checklists [43-45].

Different from our prior recommender algorithm for the inpatient setting [19], we applied a weighting factor to each query item based on its relevance to a specialty and its inverse frequency. The motivation is that inpatient clinical items are often related to acute reasons of hospitalization, while outpatient clinical items vary in scope, ranging from health maintenance or chronic disease management to treatment of urgent care issues. We show that differentially weighting query items significantly improves the performance of the recommender algorithm in both precision and recall. This makes intuitive sense because common clinical items seen in primary care clinics that are irrelevant to endocrinology likely provide less predictive power. A similar weighting scheme could be applied to other recommender algorithms when the clinical use case is specialty specific.

The association rule mining methods shown here are relatively simple to implement with interpretable results and associated statistics. Other approaches including Bayesian networks [21] and deep machine learning [46] are computationally more complex with less interpretable results. Although future research should compare these different methods, our focus primarily is to demonstrate the applicability of a data-driven approach in workup recommendations for specialty referral.

Although we ranked the recommended workup items based on PPV as shown in Table 3, we have also provided alternative metrics such as relative ratio, which could be used to look for less common but more specific or “interesting” items for a given query. For instance, in Table 3, total tri-iodothyronine had a relative ratio of 16.1, suggesting this is highly specific for patients with low TSH (indicating hyperthyroidism). In comparison, free thyroxine ranks higher based on its PPV but has a relative ratio of 1.0, suggesting this is not specific for patients with low TSH. Indeed, we observed free thyroxine also appeared in the list of recommendations for patients with high TSH (Table 3).

For a crowdsourcing clinical decision support solution like recommender algorithms, a typical concern is that recommendations drawn from common practices do not necessarily imply clinical appropriateness. To address this concern, we solicited specialist opinions on the algorithm outputs. Overall, the majority of the top recommendations were considered clinically appropriate as initial workup by the specialists. We also performed external validation by reviewing relevant guidelines, which revealed general agreement with the specialists’ assessments.

### Limitations

Limitations in this study include that the algorithm was developed at a single institution, requiring future work to expand to other institutions to evaluate generalizability. However, the algorithmic framework is general, as we used a common data model with data schema and features that were not institution specific. Second, in recommender systems such as ours, there is a well-known cold start problem when there is a lack of clinical items. Our algorithm starts with a generic “best seller list” by using the cohort item prevalence, but the algorithm could rapidly bootstrap itself with even just a couple of clinical items such as diagnosis codes or laboratory results to dynamically converge on recommendations specific to the patient scenario. Third, our cohort definition relied on referral orders placed in the electronic health records, potentially failing to capture patients who were referred to specialty clinics by other means (eg, fax or phone communication). Additionally, structured data in the electronic health records such as diagnosis codes or problem lists are often optimized for billing purpose and may be incomplete. Future research should investigate whether using unstructured text and leveraging natural language processing in clinical notes could further optimize the algorithm performance [47]. Fourth, our survey results are limited to three common referral conditions; further validation on other less common clinical conditions with more specialists from other institutions are needed. Future work should also include a prospective study to assess the effectiveness of the recommender algorithm in the specialty referral workflow. Lastly, this study did not include an analysis on the potential cost benefits of this recommender algorithm. Future research should compare the cost of additional visits due to incomplete workup with the cost of unnecessary labs if ordered based on algorithm recommendations.

### Conclusion

An item association–based recommender algorithm can predict appropriate specialist’s workup orders with high discriminatory accuracy. This could support future clinical decision support tools to increase effectiveness and access to specialty referrals. Our study demonstrates important first steps toward a data-driven paradigm for outpatient specialty consultation with a tier of automated recommendations that proactively enable initial workup that would otherwise be delayed by awaiting an in-person visit.

## Appendix

Multimedia Appendix 1 Supplemental material and table.

## Abbreviations

AUC: area under the receiver operating characteristic curve
OMOP: Observational Medical Outcomes Partnership
PPV: positive predictive value
STARR: Stanford Medicine Research Data Repository
TSH: thyroid stimulating hormone
