# Supplementary material for: A Data-Driven Algorithm to Recommend Initial Clinical Workup for Outpatient Specialty Referral: Algorithm Development and Validation Using Electronic Health Record Data and Expert Surveys
Source: JMIR Med Inform. 2022 Mar 3;10(3):e30104. doi: 10.2196/30104 (PMC8931647; doi:10.2196/30104)
Supplement: Multimedia Appendix 1 [file medinform_v10i3e30104_app1.pdf]

Supplemental Material and Table – A Data-Driven Algorithm to Recommend Initial Clinical Workup for Outpatient Specialty Referral: Algorithm Development and Validation Using Electronic Health Record Data and Expert Surveys

Table S1: 10-fold cross validation results in the training set using different weighting factors for the recommender algorithm. Weighted algorithm outperform the unweighted algorithm.

	$W_A = \frac{RR_A}{N_A}$	$W_A = \frac{1}{N_A}$	$W_A = RR_A$	$W_A = 1$
Precision (%)	52%	48%*	47%*	45% *
Recall (%)	39%	35%*	33%*	31% *
AUC	0.941	0.937*	0.935*	0.930 *
				* $P < .001$

P-value of two-tailed t-test using $W_A = \frac{RR_A}{N_A}$ *as reference.*

Specialist Survey: Please see the following pages for the survey questions in this study

Dear Colleagues,

We are working on a data-driven algorithm to recommend pre-referral laboratory workup for primary care pediatricians when they refer patients to pediatric endocrinology clinics. This survey seeks to better understand the need of pre-referral workup for the following 3 common referral reasons:

1) Abnormal thyroid studies
2) Obesity
3) Amenorrhea

The survey should take you no longer than 5 minutes to complete.

The survey is anonymous and we ask that you do not include any personally identifiable information in your responses. Your participation is voluntary. We thank you for your time to participate in this survey.

Sincerely,

Wui Ip, MD
Clinical Informatics Fellow

Priya Prahalad, MD, PhD
Clinical Assistant Professor of Pediatrics - Endocrinology and Diabetes

Jonathan Chen, MD, PhD
Assistant Professor of Medicine - Biomedical Informatics

Abnormal Thyroid Studies

Based on your experiences with patients who get referred to your clinic for abnormal thyroid studies, please answer the following questions:

What percentage of patients referred to you for abnormal thyroid studies have had appropriate pre-referral workup completed prior to their first visit with you?

> *(Note: "appropriate" means that the pre-referral workup gives you enough information to make a diagnosis and treatment recommendations)*

Please enter your estimated percentage as a whole number between 0 and 100:

[ ]

For these patients with abnormal thyroid studies, how helpful is it if they have pre-referral workup completed prior to their first visit with you?

- ○ Extremely helpful
- ○ Very helpful
- ○ Moderately helpful
- ○ Slightly helpful
- ○ Not at all helpful

Imagine a patient is referred to you for abnormal thyroid studies. Assume all you know about this patient is the referral reason and the TSH value.

For each of these studies please indicate whether it is appropriate or not appropriate as pre-referral workup based on whether the TSH is high or low.

Feel free to add additional comments in the text box below.

	If TSH is high		If TSH is low	
	Appropriate	Not Appropriate	Appropriate	Not appropriate
TSH (repeat)	○	○	○	○
Free Thyroxine	○	○	○	○
Thyroperoxidase Antibody	○	○	○	○
Thyroglobulin Antibody	○	○	○	○
Thyroid-Stimulating Immunoglobulin	○	○	○	○
Tri-iodothyronine level	○	○	○	○
Vitamin D level	○	○	○	○
Tissue transglutaminase IgA Ab	○	○	○	○
Comprehensive metabolic panel	○	○	○	○
Complete Blood Count (CBC)	○	○	○	○

Please indicate comments or other studies that you would recommend:

Obesity

Based on your experiences with patients who get referred to your clinic for obesity, please answer the following questions:

What percentage of patients referred to you for obesity have had appropriate pre-referral workup completed prior to their first visit with you?

> *(Note: "appropriate" means that the pre-referral workup gives you enough information to make a diagnosis and treatment recommendations)*

Please enter your estimated percentage as a whole number between 0 and 100:

For these patients with obesity, how helpful is it if they have pre-referral workup completed prior to their first visit with you?

- ○ Extremely helpful
- ○ Very helpful
- ○ Moderately helpful
- ○ Slightly helpful
- ○ Not at all helpful

For each of these laboratory studies please indicate whether it is

appropriate or not appropriate as pre-referral workup for a patient referred to you for obesity. Assume the referral reason is all you know about this patient.

Feel free to add additional comments in the text box below.

	Appropriate	Not Appropriate
Hemoglobin A1c	○	○
Serum glucose (include fasting)	○	○
Lipid panel (cholesterol, LDL, HDL, triglyceride)	○	○
Comprehensive metabolic panel (include LFTs)	○	○
Complete blood counts (CBC)	○	○
Vitamin D level	○	○
Free thyroxine	○	○
TSH	○	○

Please indicate comments or other labs that you would recommend for patients referred to you for obesity:

Amenorrhea

Based on your experiences with patients who get referred to your clinic for amenorrhea, please answer the following questions:

What percentage of patients referred to you for amenorrhea have had appropriate pre-referral workup completed prior to their first visit with you?

> *(Note: "appropriate" means that the pre-referral workup gives you enough information to make a diagnosis and treatment recommendations)*

Please enter your estimated percentage as a whole number between 0 and 100:

For these patients with amenorrhea, how helpful is it if they have pre-referral workup completed prior to their first visit with you?

- ○ Extremely helpful
- ○ Very helpful
- ○ Moderately helpful
- ○ Slightly helpful
- ○ Not at all helpful

For each of these laboratory studies please indicate whether it is appropriate or not appropriate as pre-referral workup for a patient referred

to you for amenorrhea. Assume all you know is the referral reason.

Feel free to add additional comments in the text box below.

	Appropriate	Not Appropriate
Testosterone level	○	○
Estradiol level	○	○
Luteinizing hormone (LH)	○	○
Follicle stimulating hormone (FSH)	○	○
17-hydroxyproesterone level	○	○
Prolactin level	○	○
DHEA sulfate level	○	○
beta-hCG level	○	○
Sex hormone binding globulin protein	○	○

Please indicate comments or other labs that you would recommend for patients referred to you for amenorrhea:

→
